# Progress of Hematopoietic Stem Cell Transplantation and Radiotherapy in the Treatment of Extranodal NK/T Cell Lymphoma

**DOI:** 10.3389/fonc.2022.832428

**Published:** 2022-02-16

**Authors:** Khodr Terro, Layal Sharrouf, Jean El Cheikh

**Affiliations:** Hematology-Oncology Division, Naef K. Basile Institiute-NKBCI, American University of Beirut, Beirut, Lebanon

**Keywords:** extranodal natural killer/t cell lymphoma, autologous hematopoietic stem cell transplantation, allogenic hematopoietic stem cell transplantation, radiotherapy, chemo radiotherapy

## Abstract

Extranodal Natural Killer/T-cell lymphoma (ENKTL) is an extremely rare type of lymphoma which is highly lethal. It mainly affects the midline area unfolding as a necrotic granulomatous and extremely disfiguring lesion. There are two subtypes of (NKTL); the most common one is nasal which appears in the nasal cavity including the nasopharynx, oropharynx, parts of the aero digestive tract and Waldeyer’s ring. While the other rarer subtype, appears in sites like the skin, testis, gastrointestinal tract, salivary glands and muscle. ENKTL is popular for the expression of multidrug resistance-associated P-glycoprotein, which not only plays the main role at exporting many antitumor agents outside tumor cells, but also makes the disease hard to treat. It is commonly associated with Epstein-Barr virus (EBV) infection and commonly occurs in Asian populations. However, there is no single unified consensus yet as to what is the standardized treatment for ENKTL. Radiotherapy alone treatment, has been considered as a first-line therapy for localized ENKTL, which later on was found to be insufficient for improving survival rates. Thus, the combination of chemotherapy and radiotherapy has been recommended as a therapeutic modality for localized ENKTL. Several combination modalities of radiotherapy and chemotherapy have been advised in clinical practice including *concurrent*, *sequential* and *sandwich chemo radiotherapy.* For the best treatment outcome, only patients with localized nasal ENKTL and low risk of treatment failure are eligible for radiotherapy. Both radiotherapy and hematopoietic stem cell transplantation (HSCT) have been used as treatment modalities in ENKTL patients. Upfront HSCT was performed for ENKTL, but it was associated with a very poor prognosis even for the limited-stage disease. The evidence supporting the use of HSCT to treat ENKTL was derived from the results of a series of phase 1 and 2 trials along with retrospective studies. The end result was a unified consensus that consolidative HSCT is not necessary in patients with newly diagnosed localized ENKTL who achieved complete response after treatment with any of the modern chemo radiotherapy regimens. Hence, HSCT is solely advised for advanced and relapsed NKTL. The main debate remains over which HSCT is the most suitable for patients with newly diagnosed advanced NKTL and relapsed NKTL.

## Introduction

Natural Killer/T-cell lymphoma (NKTL) had been previously known as lethal midline granuloma due to its aggressive nature and its preference to grow in the medial region of the face (1). Later on, it was labeled as polymorphic reticulosis since it was found to be lymphoid in origin but essentially different from the commonly known lymphomas. NKTL microscopically consisted of inflammatory cells, eosinophils and atypical lymphoid cells (2). Then it was classified in the REAL classification of lymphoid malignancies as angiocentric T-cell lymphoma due to its tendency to invade blood vessels leading to zonal necrosis (3). With the advancement of immunohistochemistry and the development of monoclonal antibodies, it was revealed that this lymphoid malignancy is NK-cell in origin. That was after detecting the cytoplasmic ϵ chain of CD3 which is characteristic of NK-cells (4). Finally, in the 2016 World Health Organization (WHO) classification of lymphoid malignancies this disease is referred to as extra nodal NK/T cell lymphoma (5). It is predominantly an extra nodal lymphoma where the nasal cavity is the most common (80%) site of involvement. The term nasal cavity includes nose, nasopharynx, oropharynx, parts of the aero digestive tract and Waldeyer’s ring. This subtype is referred to as nasal NK/T cell lymphoma (6, 7). On the other hand, the other subtypes are collectively referred to as non-nasal NK/T cell lymphoma involving sites such as the skin, testis, gastrointestinal tract, salivary glands and muscle (6). NK/T cell lymphoma can rarely develop into a disseminated and more aggressive form by spreading hematogenously, infiltrating the liver, spleen, lymph nodes and bone marrow. In this case, it would be referred to as NK/T cell leukemia (6). Extranodal NK/T cell lymphoma (ENKTL) is commonly associated with Epstein-Barr virus (EBV) infection and most commonly occurs in Asian populations (5, 8). EBV in NK/T cells was found to be clonal after terminal repeat sequences assessment. Thus, it is believed that EBV plays a crucial role in the lymphomagenesis of ENKTL, and the presence of EBV is a requirement now for the diagnosis of ENKTL along with presence of CD56 or the cytotoxic molecules such as (granzyme B, TIA1, perforin) (5). With the improvement of our understanding to the pathogenesis of ENKTL, the treatment regimen has faced drastic changes. The previously used anthracycline-containing regimens like CHOP (cyclophosphamide, doxorubicin, vincristine, and prednisolone) and CHOP-like chemotherapy combinations were found to be working poorly. This is attributed to the expression of multidrug resistance-associated P-glycoprotein, which played the role at exporting many antitumor agents outside tumor cells (9, 10). In addition, multiple studies performed discovered the benefit of Hematopoietic Stem Cell Transplantation (HSCT) and radiotherapy in the treatment of NKTL. Nevertheless, a consensus over a standardized therapy has not been established yet due to the rarity of the disease and the scarcity of prospective trials (11). In this review, we will be exploring the findings regarding the benefits of various devised protocols of HSCT and radiotherapy used in the treatment of NKTL in both the western and the eastern worlds.

## Radiotherapy in ENKTL

Different studies have shown resistance of NKTL tumor cells to anthracycline-containing combination chemotherapy, with 5-year survival rates of less than 30% (7). Since then, efforts were shifted towards studying the effectivity of radiotherapy in treating NKTL (12–14). Many retrospective analysis and prospective clinical trials were published in the Far East supporting the use of radiotherapy as the primary treatment (13, 15–18). Radiotherapy has been found to be the most important modality for treating ENKTL since the 1990s when NKTL was still referred to as angiocentric lymphoma (13). This was supported by the western studies which also appreciated the importance of radiotherapy by acknowledging the negative impact of the omission or delivery of inadequate doses of radiotherapy on the overall survival (OS) of patients with NKTL (19). Radiotherapy alone treatment has been considered as a first-line therapy for localized ENKTL. Several retrospective studies, mostly from the Far East, suggested that the appropriate radiotherapy dose in radiotherapy alone treatment of limited NKTL is 50-55 Gy (18, 20). The upfront use of radiotherapy alone has been reported to improve local control rate of localized NKTL especially the Nasal subtype (13, 21–23). Unfortunately, radiotherapy alone is found to be insufficient for improving survival due to a considerable number of patients experiencing local and systemic relapse after radiotherapy (24–26).

Thus, the combination of chemotherapy and radiotherapy became a recommended therapeutic modality for localized ENKTL (27). Several combination modalities of radiotherapy and chemotherapy have been advised in clinical practice including *concurrent*, *sequential* and *sandwich chemo radiotherapy* (28). Technically, those three modalities stemmed from the ongoing debate of whether to employ radiotherapy as an upfront therapy or as a sequential therapy preceded by chemotherapy in limited stage ENKTL. Employing radiotherapy as an upfront therapy, achieves high treatment response rates and reduces the risk of chemo resistance. However, initiating chemotherapy followed by radiotherapy has shown to reduce the risk of systemic failure and distant metastasis (28).

Starting with *concurrent chemo radiotherapy*, a phase II trial in Japan tested the 3 cycles DeVIC (dexamethasone, etoposide, ifosfamide, and carboplatin) chemotherapy regimen simultaneously with 50 Gy radiotherapy over a period of 6-8 weeks (**Table 1**). It has shown a complete response (CR) rate of 77%, an overall response (OR) rate of 81%, 5-year OR of 70% and a 5-year progression free survival (PFS) of 63% (17, 29). Despite this regimen positive results, it has shown that the simultaneous administration of chemotherapy and radiotherapy increases the risk of hematological and non-hematological toxicities regardless of chemotherapy regimen. Therefore, changing the chemotherapy regimen to other regimens such as ESHAP (etoposide, steroid, high-dose Ara-C and cisplatin) and DEP (dexamethasone, etoposide and cisplatin) did not reduce toxicity rates (16, 30). Hence, another *concurrent chemo radiotherapy regimen* emerged consisting of 4 weeks of radiotherapy + cisplatin followed by an adjunct 3 cycles of VIPD (etoposide, ifosfamide, cisplatin, and dexamethasone), showed a CR rate of 80% and a 3-year PFS of 85%. In this regimen, the radiation dose has been reduced to around 40 Gy due to the radio-sensitizing quality of cisplatin. Many similar regimens have been employed in various trials with pretty close outcomes, but all have shown a significant potential risk of systemic disease progression during the period of radiotherapy (16, 31, 32). The latter issue led to two other chemo radiotherapy regimens (*sequential* and *sandwich* chemo radiotherapy) which start with chemotherapy as an upfront therapy.

**Table 1:** Radiotherapy in NKTL.

RADIOTHERAPY REGIMENS	Indications	References	PFS	OS	CR
***Radiotherapy alone***	-Limited stage ENKTL	(23)	68.5%	77%	73.7%
***Concurrent Chemoradiotherapy***	-Elderly -Low tumor burden (stage I) -Normal LDH -Absence of primary tumor invasion -Nasal NKTL with low risk	(29)	63%	81%	77%
DeVIC (dexamethasone, etoposide, ifosfamide, and carboplatin) chemotherapy regimen + (50 Gy) radiotherapy over a period of 6-8 weeks					
***Concurrent Chemoradiotherapy***	–	(41)	85%	–	80%
4 weeks of 40 Gy radiotherapy + cisplatin followed by an adjunct 3 cycles of VIPD (etoposide, ifosfamide, cisplatin, and dexamethasone)					
***Sequential Chemoradiotherapy***	-Nasal NKTL with intermediate risk	(6)	–	90%	69%
SMILE (dexamethasone, methotrexate, ifosfamide, L-asparaginase, etoposide)+RT (50Gy)					
***Sandwich Chemoradiotherapy***	-Nasal NKTL with low risk	(35)	74%	96%	74%
GELOX (gemcitabine, oxaliplatin and asparaginase) + sandwiched RT (56 Gy)					

The *sequential chemo radiotherapy* with 2-4 cycles of SMILE (dexamethasone, methotrexate, ifosfamide, L-asparaginase, and etoposide) followed by radiotherapy was demonstrated in a phase II clinical trial on stage IV NKTL. The results portrayed a CR rate of 69% and an OR rate of 90% (33). The SMILE has shown to commonly inflict severe hematologic toxicities which were intolerable in elderly and frail patients (6). Therefore, this therapy was advised as a treatment regimen for young low comorbid patients with localized nasal NKTL (6).

Finally, concerning the *sandwich chemo radiotherapy* regimen, a phase II trial has studied the employment of 2 cycles of GELOX (gemcitabine, L-asparaginase, and oxaliplatin) followed by radiotherapy of 56 Gy, then after a week 2-4 cycles of GELOX were added. Patients who experienced hypersensitivity to L-asparaginase were given pegaspargase instead. The outcome of this trial has shown a CR rate of 74% and an OR rate of 96% with an 85% 5-year PFS and 74% 5-year OS (34, 35). Despite the fact that this regimen is the most time consuming, it has shown the least levels of toxicities rendering it the most suitable for localized nasal NKTL in elderly and frail patients (36, 37).

More efforts have been exerted to decrease the toxicities induced by radiotherapy through implementing intensity modulated radiotherapy (IMRT) and volumetric modulated arc therapy (VMAT). Both of which are designed to use different types of boluses providing the patients with higher individuation and improved conformity and uniformity of the planning target volume. In other words, all these efforts are striving to make radiotherapy more effective which would permit the decrease of doses received by patients and consequently decrease the toxicity levels due to radiotherapy (37–40).

Furthermore, in order to achieve the best treatment outcome, radiotherapy should only be offered to patients with localized nasal ENKTL and low risk of treatment failure. Hence, a risk evaluation index (prognostic index of natural killer lymphoma (PINK)) was proposed to help identify people with advanced disease where radiotherapy is not effective (41).

## Hematopoietic Stem Cell Transplantation in ENKTL

Both upfront *autologous and allogenic HSCT* were performed for NKTL when the disease had not yet received its finalized nomenclature and was associated with poor prognosis even for the limited-disease stage (42). The evidence supporting the use of HSCT to treat ENKTL was derived from the results of a series of phase 1 and 2 trials along with retrospective studies. The studies reached a unified consensus that consolidative HSCT is not necessary in patients with newly diagnosed localized ENKTL who achieved CR after treatment with any of the modern chemo radiotherapy regimens previously discussed (29, 43, 44). The main debate remains over which HSCT is the most suitable for patients with newly diagnosed advanced NKTL and relapsed NKTL.

In a retrospective study for 62 patients, 50% of those had advanced stage NKTL and most of them received non-anthracycline-containing induction regimen. Around 65% had achieved CR pre *auto-HSCT*. After a 3-year follow-up the PFS and OS the came out to be 52.4% and 60% respectively. Patients with limited disease for sure did significantly better than the ones with advanced disease. Although 90% of patients with advanced NKTL were treated with non-anthracycline containing induction regimens, the 3-year PFS and OS came out to be 40.1% and 52.3% respectively. Unfortunately, all this did not show any difference compared to patients not receiving transplant from the previous studies (11). Another retrospective study from China had 20 patients mostly with advanced ENKTL who underwent upfront *auto-HSCT* following induction therapy with L-asparaginase-containing-chemotherapy with or without radiotherapy. The outcome of this group was compared to a control group consisting of 60 patients mostly with advanced ENKTL receiving the same chemotherapy and were eligible for *auto-HSCT*, but they declined. The 5-year OS of the *auto-HSCT* group came out to be 79.3% which is comparably higher than that of the control group which was 52.3%. While *auto-HSCT* group didn’t show a statistical significance in increasing the PFS rate compared to the control group (P=0.079). Based on the results of this retrospective study, the disease status and staging were found to be an important variable in giving the final verdict about the benefit of *auto-HSCT* in NKTL. Even though the *auto-HSCT* group showed a better OS than the control group, there was no additional benefit seen when comparing the outcomes of stage I/II NKTL patients from both groups (45). The European Society for Blood and Marrow Transplantation (EBMT) had a cohort of 28 patients who underwent *auto-HSCT*. The 2-year PFS and OS rates of the advanced-stage NKTL patients were 41% and 52% respectively. The outcome of those *auto-HSCT* patients, who received L-asparaginase-containing-induction therapy, was comparable to the outcomes in other similar studies (46). As a result, an official consensus was released by the American Society of Blood and Marrow Transplantation (ASBMT) to reserve *auto-HSCT* only for advanced-stage, relapsed or disseminated NKTL (43).

On the other hand, *allo-HSCT* has been evaluated in a retrospective analysis of 36 patients treated for NKTL where 10 patients were at an advanced stage. The 2-year PFS and OS were 30% and 40% respectively (**Table 2**). Most of the patients received L-asparaginase-containing induction regimen pre *allo-HSCT.* The main disadvantageous factors that played a role in decreasing OS rates were relapse, GVHD and infection (47). Another retrospective study, with patients receiving L-asparaginase-containing induction regimen pre *allo-HSCT* as well, was led by Center for International Bone Marrow Transplant Registry (CIBMTR) on 25 patients with 21 of them diagnosed with advanced stage NKTL. The 2-year PFS and OS rates were reported to be 20% and 24% respectively. The CR pre *allo-HSCT* was found to be the main predictor in achieving better survival rates (P< 0.001) (43). A multicenter retrospective analysis, led by the Asian Lymphoma Study Group with a total of 18 patients, studied *allo-HSCT* survival rates following L-asparaginase induction therapy as well. The 5-year PFS and OS were 51% and 57% respectively. It was noted that the use of SMILE as an induction regimen pre *allo-HSCT* showed a significant improvement in both PFS and OS. However, it should be noted that the last conclusion was made based on a limited cohort of patients. In this retrospective study both non-relapse-mortality and infection were the major contributors to mortality (48). Both infection and GVHD remain as main limitations in *allo-HSCT*, but multiple studies are being devised to improve this disadvantageous aspect of *allo-HSCT*. A very recent retrospective Spanish study, over a period of 25 years with a cohort of 201 patient undergone *allo-HSCT* for either NKTL or T-cell neoplasia, has shown that both post-transplant cyclophosphamide (PTCy) and haploidentical donor significantly decreased the risk for acute GVHD (49). Moreover, the Japanese Journal of Prosthodontic Research has highlighted in their very recent case report the importance of providing dental care with customized care plan before, during, and after HSCT for NKTL. The latter is based on their case report which demonstrated a significant decrease in risks of infections and oral function complications (50). As a result, the ASBMT reached a consensus on *allo-HSCT* in NKTL as well. They recommend *allo-HSCT* for disseminated NKTL in 1^st^ CR, for localized NKTL with chemo sensitive relapsed disease, and for localized NKTL with primary refractory or relapsed/refractory disease (43).

**Table 2:** HSCT in NKTL.

HSCT	Indications	References	TRM	GVHD	PFS	OS
**Autologous**	-Advanced-stage, relapsed -Disseminated NKTL	(11)	–	–	52.4%	60%
		(45)	–	–	60%	79%
		(46)	–	–	41%	52%
**Allogenic**	-Disseminated NKTL in 1st CR -Localized NKTL with chemosensitive relapsed disease -Localized NKTL with primary refractory or relapsed/refractory disease	(47)	30%	11%	30%	40%
		(48).	5%	50%	51%	57%
		(43)	–	–	20%	24%

## Conclusion

In conclusion, the prognosis of NKTL has never been any better than today. The role of radiotherapy and HSCT continue to evolve and improve. As for radiotherapy, the combination of chemotherapy (non-anthracycline, platinum or L-asparaginase containing) and radiotherapy is now advised as a therapeutic modality for only localized ENKTL with low risk of treatment failure. As for HSCT, it should be reserved for advanced stages of NKTL. Both *auto-HSCT* and *allo-HSCT* have shown promising results in advanced NKTL patients. *Auto-HSCT* is preferred more for disseminated NKTL with 1^st^ CR, while *allo-HSCT* for refractory NKTL. Moreover, multiple studies are being devised to improve the two main limitations of HSCT which are GVHD and infection. For us to better understand and treat such a highly fatal and rare disease, additional multi-institutional collaborations and prospective clinical trials are needed.

## Author Contributions

JC is the corresponding author. KT is the first author responsible for writing the manuscript. LS is the second author responsible for editing and finalizing. All authors contributed to the article and approved the submitted version.

## Conflict of Interest

The authors declare that the research was conducted in the absence of any commercial or financial relationships that could be construed as a potential conflict of interest.

## Publisher’s Note

All claims expressed in this article are solely those of the authors and do not necessarily represent those of their affiliated organizations, or those of the publisher, the editors and the reviewers. Any product that may be evaluated in this article, or claim that may be made by its manufacturer, is not guaranteed or endorsed by the publisher.
